# Cerebral ventriculomegaly in myotonic dystrophy type 1: normal pressure hydrocephalus-like appearances on magnetic resonance imaging

**DOI:** 10.1186/s12868-021-00667-8

**Published:** 2021-10-18

**Authors:** Saya Iida, Hiroko Seino, Fumiko Nagahata, Soichiro Tatsuo, Sho Maruyama, Seiko Kon, Hiroto Takada, Masashi Matsuzaka, Koichiro Sugimoto, Shingo Kakeda

**Affiliations:** 1 Department of Radiology, Hirosaki University Graduate School of Medicine, 5 Zaifu-cho, Hirosaki, Aomori 036-8562 Japan; 2 Department of Radiology, National Hospital Organization, Aomori Hospital, Aomori, Aomori Japan; 3 Department of Neurology, National Hospital Organization, Aomori Hospital, Aomori, Aomori Japan; 4 Clinical Research Support Center, Hirosaki University Hospital, Hirosaki, Aomori, Japan; 5 Department of Medical Informatics, Hirosaki University Hospital, Hirosaki, Aomori Japan; 6 Department of Radiology, University of Occupational and Environmental Health, School of Medicine, Kitakyushu, Japan

**Keywords:** Myotonic dystrophy, ventriculomegaly, Evans Index, Normal pressure hydrocephalus, Disproportionately enlarged subarachnoid-space hydrocephalus, MRI

## Abstract

**Background:**

Cerebral ventriculomegaly is an abnormal feature characteristic of myotonic dystrophy type 1 (DM1). This retrospective study investigated the morphologic changes accompanied by ventriculomegaly in DM1 on brain MRI.

**Methods:**

One hundred and twelve adult patients with DM1 and 50 sex- and age-matched controls were assessed. The imaging characteristics for evaluations included the z-Evans Index (ventriculomegaly), callosal angle (CA), enlarged perivascular spaces in the centrum semiovale (CS-EPVS), temporo-polar white matter lesion (WML) on 3D fluid-attenuated inversion recovery (FLAIR), disproportionately enlarged subarachnoid-space hydrocephalus (DESH), and pathological brain atrophy. The “z-Evans Index” was defined as the maximum z-axial length of the frontal horns to the maximum cranial z-axial length. To determine the imaging characteristics and genetic information (CTG repeat numbers) that were associated with the z-Evans Index, we used binominal logistic regression analyses.

**Results:**

The z-Evans Index was significantly larger in the patients than in the controls (0.30 ± 0.05 vs. 0.24 ± 0.02; p < 0.01). The z-Evans Index was independently associated with the callosal angle (p < 0.01) and pathological brain atrophy (p < 0.01) but not with age, gender, CTG repeat numbers, or CS-EPVS. Of the 34 patients older than 49 years, 7 (20.6%) were considered to have DESH.

**Conclusions:**

Our MRI study revealed a normal pressure hydrocephalus (NPH)-like appearance as a morphologic finding accompanied by ventriculomegaly in DM1 that tends to occur in elderly patients.

## Background

Myotonic dystrophy type 1 (DM1) is the most common disease that can cause muscle weakness and atrophy among adults [1]. The genetic defect underlying DM1 is multiple CTG repeats in the 3' untranslated region of a gene (DM1) on the long arm of the chromosome: the analysis of the CTG repeat on chromosome 19q13 [2]. Neuropathological, neuroimaging, and neurophysiological studies have demonstrated a variety of abnormalities of the central nervous system (CNS) in DM1 patients [3–5]. Magnetic resonance imaging (MRI) has been widely used to evaluate DM1. Previous MRI studies with DM1 demonstrated ventriculomegaly and "unusual" white matter lesions (WMLs) in the anterior or medial portions of the temporal lobes (temporo-polar WMLs) [6]. Enlarged perivascular spaces (EPVS) on MRI were also seen in the centrum semiovale (CS) and basal ganglia in DM1 [7].

Normal pressure hydrocephalus (NPH) is a syndrome of gait dysfunction and enlarged cerebral ventricles in the absence of another cause. It is frequently accompanied by dementia and bladder detrusor overactivity [8, 9]. The National Institutes of Health (NIH) (https://www.ninds.nih.gov/) reports that, in patients with suspected NPH, a variety of tests, including brain CT and/or MRI, a spinal tap or lumbar catheter, intracranial pressure monitoring, and neuropsychological tests are helpful to diagnose NPH and rule out other conditions. The study of NPH on Neurologic Improvement (SINPHONI) described important MRI features for the NPH diagnosis [10] as high-convexity tightness and an enlarged Sylvian fissure with ventriculomegaly, designated as "disproportionately enlarged subarachnoid-space hydrocephalus (DESH)." The Evans Index has also been the most popular index of ventriculomegaly. Through our work, we have noticed that the MRI features specified in NPH (NPH-like appearance = DESH) are frequently seen in DM1 patients.

Ventriculomegaly is a characteristic MRI finding in DM1 [11]. However, to our knowledge, no previous studies have evaluated the morphologic MRI changes accompanied by ventriculomegaly in DM1. Furthermore, no previous studies have mentioned “NPH-like appearances” in DM1 patients, although Wang et al. demonstrated the coexistence of DM1 and NPH, as five patients were available for case reviews [12]. The present study therefore investigated the frequency of an NPH-like appearance in DM1 patients. We also determined whether or not an NPH-like appearance was associated with the clinical characteristics and previously described typical DM1 features on brain MRI.

## Methods

### Subjects

The study was conducted in accordance with the Helsinki Declaration and Good Clinical Practice. The ethics review committees of Aomori Hospital approved the study (approval number: 1–18). The ethics review committees approved this retrospective study, and the need to obtain informed consent from the patients was waived.

We routinely perform screening brain MRI studies to assess DM1 patients. One radiologist (FN with 4 years of neuroradiology experience) reviewed a clinical database between April 2018 and May 2020 and selected adult 112 patients who had been diagnosed with DM1 based on a clinical examination, electromyography, and underlying genetic defects (the analysis of the CTG repeat on chromosome 19q13 by standard techniques [13]). All 112 patients had undergone brain MRI. No patients were excluded based on the exclusion criteria, including patients with a history of other neurological diseases and/or unsatisfactory images due to artifacts (Table 1).

**Table 1:** Clinical and imaging characteristics of DM1 and controls

		DM1 (n = 112)	Controls (n = 50)	p value
Clinical characteristics	Age, mean (IQR)	44.3 (36–53)	45.7 (38–53)	0.49^a^
	Sex, female (%)	48 (43)	26 (52)	0.31^b^
DM1 related factors	CTG repeat numbers, mean (IQR)	950 (600–1366)	NA	NA
Imaging characteristics	z-Evans Index, mean (IQR)	0.29 (0.27–0.32)	0.24 (0.23–0.26)	< .01^a^
	Callosal angle, mean (IQR)	123 (115–129)	123 (112–127)	0.83^a^
	Sylvian fissure dilation, n (%)	61 (54)	2 (4)	< .01^b^
	High-convexity tightness, n (%)	9 (8)	0 (0)	0.06^b^
	CS-EPVS, n (%)	35 (31)	9 (18)	0.09^b^
	Temporo-polar WMH, n (%)	82 (73)	0 (0)	< .01^b^
	Pathological brain atrophy, n (%)	46 (41)	1 (2)	< .01^b^

*DM1* = myotonic dystrophy type 1, *IQR *interquartile range, *NA* not applicable, *CS-EPVS* enlarged perivascular spaces in centrum semiovale; temporo-polar, *WML *temporo-polar white matter lesion

^a^Mann–Whitney U test

^b^Fisher’s exact test

We selected 50 age- and sex-matched controls from the same sample population who had no history of neurological or psychiatric diseases and who had undergone MRI. The indications for their examinations included screening, headaches, or benign positional vertigo. By reviewing the neuroradiologists (HS and SKa with 12 and 24 years of experience in neuroradiology, respectively), the conventional MRI results were normal in all controls.

### Image acquisition

All studies were performed using a 1.5-T scanner (Magnetom Symphony, Syngo Version VE11C; Siemens Medical Systems, Erlangen, Germany) equipped with gradients with maximal gradient strength, 40 mTm − 1 and maximal slew rate, 200 mTm − 1/ms using a 20-channel head coil. All patients and controls underwent brain MRI according to our standard protocol, including axial T2-weighted imaging (T2WI), two-dimensional (2D) coronal fluid-attenuated inversion recovery (FLAIR) imaging, and three-dimensional (2D) sagittal FLAIR. For 3D FLAIR (3D fast spoiled gradient-echo imaging), we used three cross-section images (coronal, axial, and sagittal) reconstructed from the images obtained on the sagittal plane**.** The imaging parameters (repetition time ms/echo time ms/inversion time/NEX/echo train length/matrix/field of view/imaging time) were 4,000/85/NA/2/9/226 × 384/23 cm/2 min and 12 s for 2D T2WI and 9,000/108/2,500/1/14/208 × 320/22 cm/3 min and 18 s for 2D FLAIR imaging, respectively. The 2D T2WI and 2D FLAIR images were acquired at a section thickness of 5 mm, an intersection gap of 1.0 mm. For 3D FLAIR, the following imaging parameters were used: 5,000/333/1800/1/235/248 × 256/24 cm/1.0 mm/2/5 min and 17 s (repetition time ms/echo time ms/inversion time/ NEX/echo train length/matrix/field of view/section thickness/ reduction factor/imaging time, respectively).

### Image interpretation

#### Quantitative assessments

According to the previous method [14, 15], quantitative MRI evaluations were performed by one radiologist (SI with 5 years of experience in neuroradiology).

**Fig. 1:**
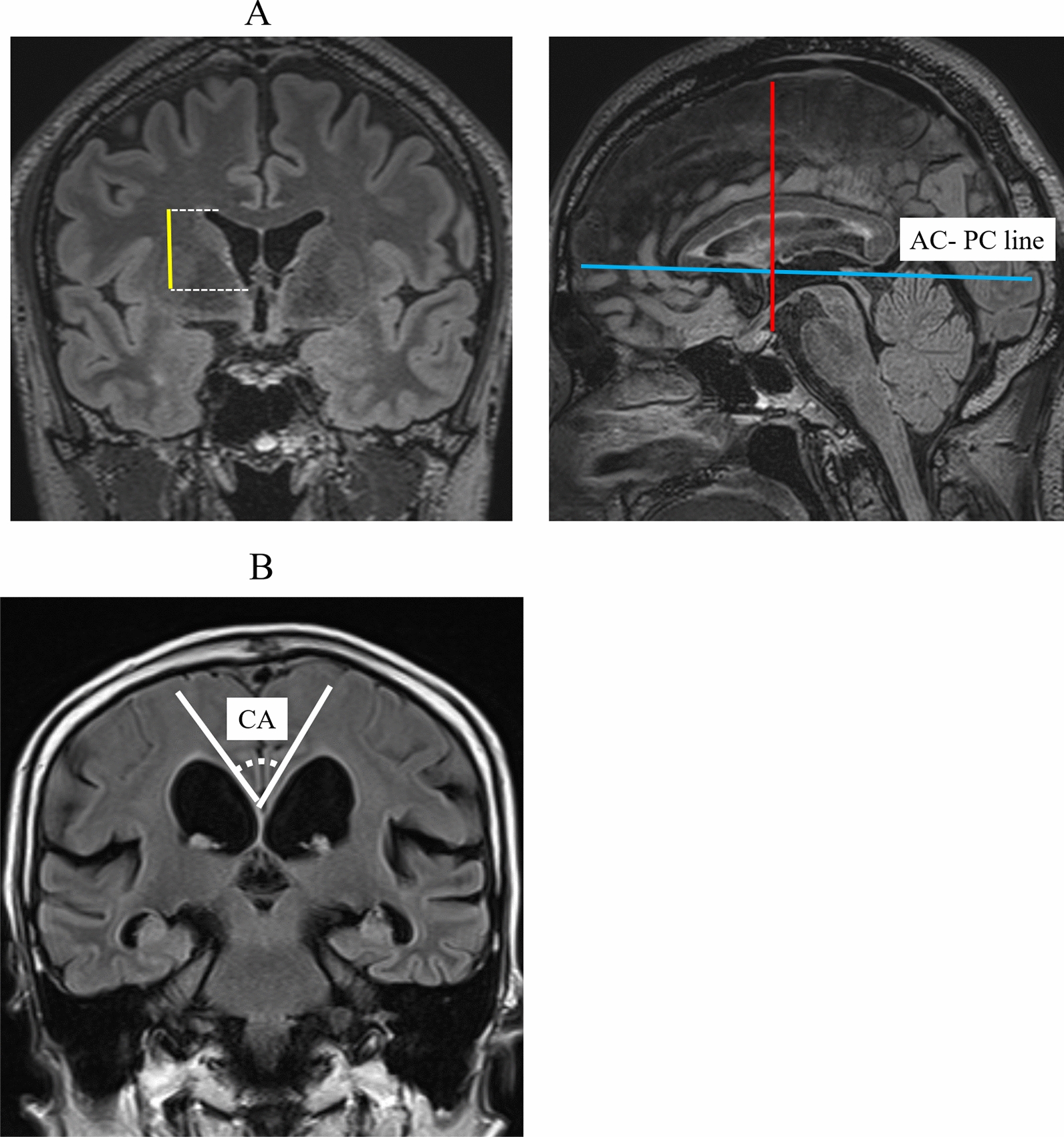
** A** z-Evans Index and Callosal angle.** A** The figures are the MPR reconstructions of 3D FLAIR images. The z-Evans Index is defined as the maximum length (yellow lines) from the foramen of Monro to the superior margin of the frontal horns (dashed lines) on coronal FLAIR images / the maximum cranial z-axial length (red lines) on sagittal FLAIR images. The blue line indicates the anterior and posterior commissure (AC- PC) line.** B** Callosal angle

In the current study, on the coronal and sagittal FLAIR images, the radiologist measured the z-Evans Index, an alternative to the Evans Index. Yamada et al. found that, by using quantitative volumetric analyses, the volume expansion of the bilateral ventricles, especially at the frontal horns, was toward the z-axial direction, rather than the x-axial direction, in patients with NPH [14]. They therefore newly proposed the z-Evans Index, which was a representative index for z-axial directional expansion of the frontal horns of the bilateral ventricles. The "z-Evans Index" was defined as the maximum z-axial length of the frontal horns to the maximum cranial z-axial length (Fig. 1). The radiologist also used FLAIR images to measure the callosal angle (Fig. 1), which is the angle between the lateral ventricles on a coronal image through the posterior commissure, perpendicular to the anterior/posterior commissure plane [15].

#### Qualitative assessments

Two certified neuroradiologists (HS and SKa with 12 and 24 years of experience in neuroradiology, respectively) who did not take part in the image processing independently reviewed the MRI scans and categorized the findings (Sylvian fissure dilation, high-convexity tightness, CS-EPVS, temporo-polar WML, and brain atrophy) by consensus.

**Fig. 2:**
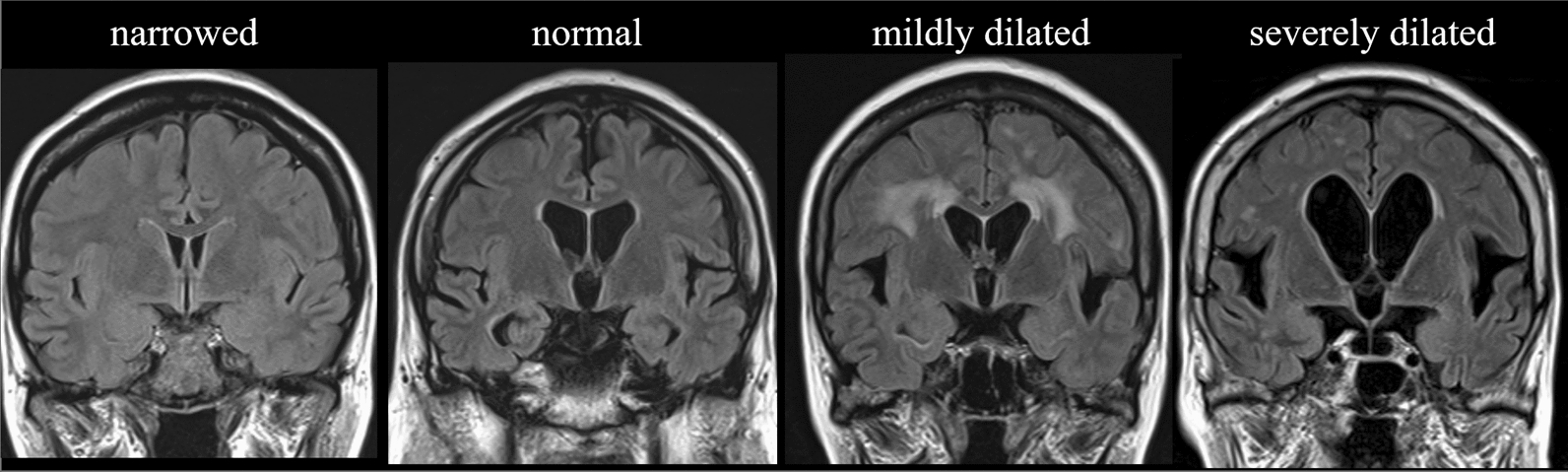
Classification for Sylvian fissure dilation. Visual rating scales for Sylvian fissure dilation coronal FLAIR images: 1, narrowed or normal; 2, mildly dilated (abnormal dilation of Sylvian fissure); and 3, severely dilated (abnormal dilation of Sylvian fissure with widely-detachment of frontal and temporal opercula from the insular cortex)

**Fig. 3:**
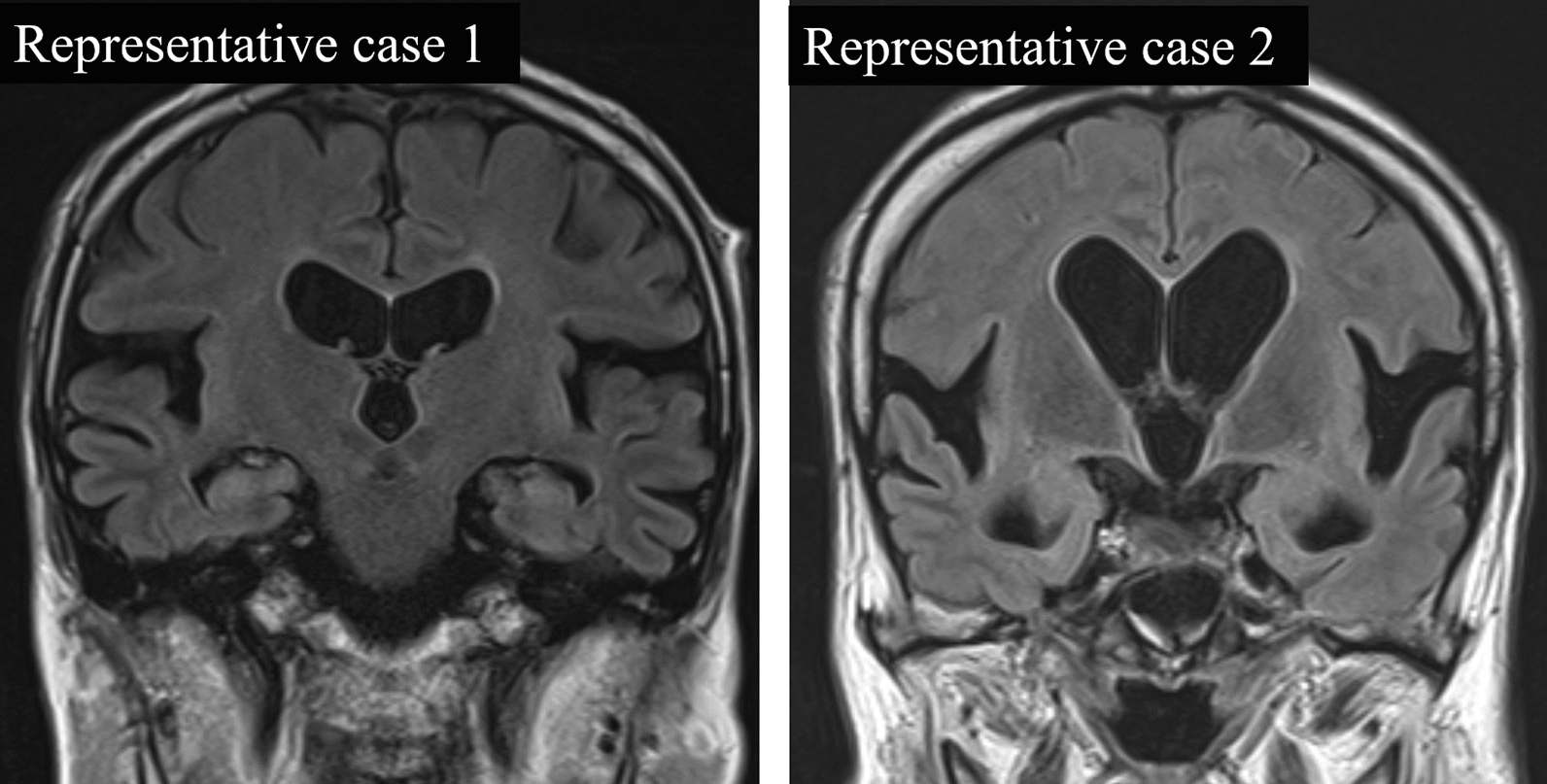
Classification for Sylvian fissure dilatation and high convexity tightness. Representative case 1 is rated as “normal” for absence of the high-convexity tightness and “severely dilated” for the Sylvian fissure dilation. Representative case 2 is rated as “extremely tight” for the high-convexity tightness and “severely dilated” for the Sylvian fissure dilation. Therefore, Representative case 2 shows the findings of disproportionately enlarged subarachnoid space hydrocephalus (DESH)

The width of the Sylvian fissure was assessed on transverse and coronal sections. We used the following visual rating scale: 1, narrowed or normal; 2, mildly dilated (abnormal dilation of Sylvian fissure); and 3, severely dilated (abnormal dilation of Sylvian fissure with widely-detachment of frontal and temporal opercula from the insular cortex) on the coronal images [16] (Fig. 2). We evaluated the high-convexity tightness on the four uppermost contiguous transverse sections and three contiguous coronal sections on and anterior to the posterior commissure. The severity of the high-convexity tightness was visually rated as follows: 1, dilated or normal; 2, mildly tight (tightness was observed over less than three-quarters of the high-convexity space); and 3, extremely tight (tightness was observed over three-quarters or more of the high-convexity space) (Fig. 3).

According to the previous reports [17], on T2WI, the radiologists assessed the presence of EPVS in the CS (CS-EPVS: identified along the perforating medullary arteries as they penetrated the cortical gray matter over the high convexities and extended into the white matter). The CS-EPVS were coded with the following scale (which was applied to standard axial images): 0 = no EPVS, 1 = 1 to 10 EPVS, 2 = 11 to 20 EPVS, 3 = 21 to 40 EPVS, and 4 =  > 40 EPVS). The radiologists also evaluated the 3D FLAIR images for the presence or absence of temporo-polar WMLs. Brain atrophy was defined as enlargement of the gyri and rated on a subjective scale of 0 to 3 (0 = absent, 1 = mild, 2 = moderate, 3 = severe)[18].

### Statistical analyses

For the analyses, the age, CTG repeat numbers, z-Evans Index, and callosal angle were treated as continuous variables, while the sex, high convexity tightness, Sylvian fissure dilation, CS-EPVS, and temporo-polar WMLs were treated as categorical variables.

A z-Evans Index of > 0.3 was arbitrarily defined as ventriculomegaly. For the subjective scale of high-convexity tightness and Sylvian fissure dilation, the “2 or 3” were defined as pathologic findings. Thus, high-convexity tightness (2 or 3) combined with Sylvian fissure dilation (2 or 3) accompanied by ventriculomegaly was defined as DESH (Fig. 3: Representative case 2). We defined high CS-EPVS (3 or 4) as the presence of > 20 EPVS, in line with the most severe category of EPVS used in previous studies [17, 19]. For the subjective scale of brain atrophy, “2 or 3” was defined as pathological brain atrophy.

We compared the clinical and imaging characteristics between patients and controls using Fisher’s exact test for categorical variables and the Mann–Whitney U test for continuous variables, as appropriate.

To determine the imaging characteristics (callosal angle, Sylvian fissure dilation, high-convexity tightness, CS-EPVS, temporo-polar WML, or pathological brain atrophy), genetic information (CTG repeat numbers), and the patient age and gender that were associated with the z-Evans Index (ventriculomegaly), we conducted the binominal logistic regression analyses. The analyses were performed by excluding the finding of “Sylvian fissure dilation”, “high-convexity tightness”, and “temporo-polar WML”, and because these findings are obvious confounding factors.

We arbitrarily divided the DM1 patients into three age groups: ≤ 39, 40–49, and ≥ 50 years old (Age-group A, B, and C, respectively) by considering our sample size and distribution. Because the continuous variables in this study showed non-normal distribution, we compared the clinical and imaging characteristics among the three age groups using the Steel–Dwass test for continuous variables and Ryan's procedure for categorical variables. All analyses, *P* values < 0.05 were considered indicate statistical significance. All of the statistical analyses were performed using the R software program [20].

## Results

Table 1 summarizes the clinical and radiological characteristics of the DM1 patients and controls. The z-Evans Index was significantly larger in the patients than in the controls (0.30 ± 0.05 vs. 0.24 ± 0.02; p < 0.01). The Sylvian fissure dilation (54 vs. 4%; p < 0.01), temporo-polar WML (73 vs. 0%; p < 0.01), and pathological brain atrophy (41 vs. 2%; p < 0.01) were more commonly seen in the patients than in the controls.

Table 2 lists the results of the logistic regression analyses for the z-Evans Index. The z-Evans Index was independently associated with the callosal angle (p < 0.01) and pathological brain atrophy (p < 0.01) but not with age, gender, CTG repeat numbers, or CS-EPVS.

**Table 2:** The binominal logistic regression analyses for the Z-Evans Index in DM1 patients

			AOR (95% CI)	p value
Clinical characteristics	Age		1.030 (0.98–1.08)	0.21
	Gender	Male	1.00	0.80
		Female	0.886 (0.35–2.27)	
DM1 related factors	CTG repeat numbers		1.000 (1.00–1.00)	0.55
Imaging characteristics	Callosal angle		0.944 (0.91–0.98)	< 0.01
	CS-EPVS	( +)	1.00	0.08
		(−)	0.442 (0.15–1.28)	
	Pathological brain atrophy	( +)	1.00	< 0.01
		(−)	4.610 (1.71–12.4)	

*AOR*  adjusted odds ratio, *CI* confidence interval, *DM1* myotonic dystrophy type 1, *CS-EPVS* enlarged perivascular spaces in centrum semiovale

**Fig. 4:**
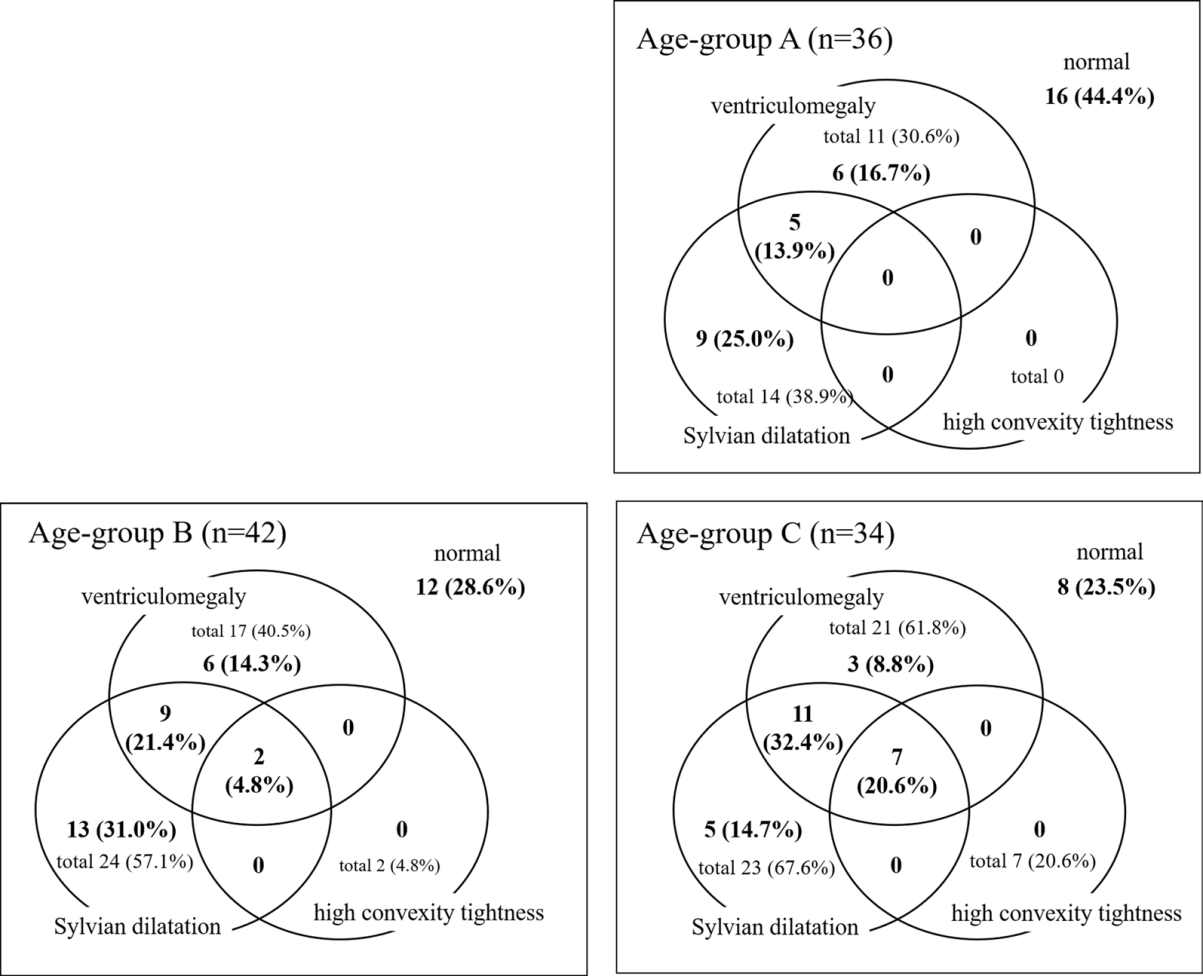
The proportion of imaging features for each age group of DM1 patients. Age-group A: ≤ 39 years old. Age-group B: 40–49 years old. Age-group C: ≥ 50 years old. A z-Evans Index of > 0.3 was defined as ventriculomegaly. For the subjective scale of high-convexity tightness and Sylvian fissure dilation, “2 or 3” was considered a pathologic finding. Thus, high-convexity tightness (2 or 3) combined with Sylvian fissure dilation (2 or 3) accompanied by ventriculomegaly was defined as disproportionately enlarged subarachnoid space hydrocephalus (DESH)

Figure 4 shows the proportion of imaging features for each age group of DM1 patients. As shown in Age-group C in Fig. 4, ventriculomegaly was observed in 21 (61.8%) of 34 patients. Most (n = 18) of the 21 patients with ventriculomegaly in Age-group C showed Sylvian fissure dilation, whereas only 5 of the 11 patients with ventriculomegaly in Age-group A showed it. In Age-group C, high-convexity tightness was found in 7 patients (20.6%), all of whom had ventriculomegaly as well as Sylvian fissure dilation and were thus diagnosed with DESH. No and two patients were diagnosed with DESH in Age-group A and B, respectively.

Table 3 summarizes the clinical and imaging characteristics for the age group comparisons in DM1 patients. The z-Evans Index was significantly higher in Age-group C than in Age-group A (p < 0.01) or B (p < 0.05). The presence of the DESH was more commonly seen in Age-group C than in Age-group A (p < 0.01). With regard to the other imaging characteristics (callosal angle, CS-EPVS, temporo-polar WMLs, and pathological brain atrophy), there were no statistically significant differences among the three age groups.

**Table 3:** Findings according to the age of DM1 patients

		Age-group A ≤ 39 years old (n = 36)	Age-group B 40–49 years old (n = 42)	Age-group C ≥ 50 years old (n = 34)	Age-group A vs B	Age-group A vs C	Age-group B vs C
Clinical characteristics	Age, mean (IQR)	33.5 (29.5–36)	45 (42–47)	55.5 (53–61)	NA	NA	NA
	Sex, female (%)	16 (44)	15 (36)	17 (50)	NS	NS	NS
DM related factors	CTG repeat numbers, mean (IQR)	1100 (600–1450)	950 (700–1200)	833 (550–1200)	NS	NS	NS
Imaging characteristics	z-Evans Index, mean (IQR)	0.282 (0.250–0.303)	0.291 (0.278–0.313)	0.313 (0.283–0.374)	NS	*	**
	Callosal angle, mean (IQR)	123 (119–129)	123 (112–129)	125 (103–129)	NS	NS	NS
	DESH, n (%)	0 (0)	2 (5)	7 (21)	NS	*	NS
	CS-EPVS, n (%)	17 (47)	10 (24)	8 (24)	NS	NS	NS
	Temporo-polar WML, n (%)	25 (69)	30 (71)	27 (79)	NS	NS	NS
	Pathological brain atrophy, n (%)	13 (36)	21 (50)	12 (35)	NS	NS	NS

*DM1*  myotonic dystrophy type 1, *IQR*  interquartile range, *NA*  not applicable, *NS* not significant, *DESH *disproportionately enlarged subarachnoid space hydrocephalus, *CS-EPVS* enlarged perivascular spaces in centrum semiovale, *temporo-polar WML * temporo-polar white matter lesion

*Significantly different (p < 0.01)

**Significantly different (p < 0.05)

## Discussion

In the present study, we evaluated DM1 patients using the newly proposed z-Evans Index. Although the original Evans Index has been adopted as a criterion for discriminating the tap-test response [21], the z-Evans Index has been shown to have a higher diagnostic accuracy [14]. We found that the z-Evans Index of DM1 was significantly higher than that of control patients. Furthermore, in our study, the DESH was observed in 20.6% of elderly patients with DM1. These results were consistent with those of previous studies showing that cerebral ventriculomegaly was common in DM1 patients and appeared to be progressive [11].

It is important to note that an increased z-Evans Index (ventriculomegaly) was independently associated with the callosal angle. The callosal angle is a specific quantitative imaging biomarker in the diagnosis of NPH, allowing improved differentiation from other neurodegenerative disorders with ventriculomegaly, prediction of surgical shunt responsiveness, and sensitivity to ventricular changes following surgical shunt [22–27]. Another study showed a significantly decreased mean callosal angle in patients with NPH (109 ± 9) compared with patients with Alzheimer’s disease (AD) (135.4 ± 11.3) or dementia with Lewy bodies (DLB**)** (136.9 ± 8.2) [28]. The mean callosal angle (113 ± 26.4°) in our elderly patients (Age-group C) (Table 3), which might be close to that in NPH, was smaller than that reported in AD and DLB cases [28]. These results suggest that the morphologic changes accompanied by ventriculomegaly in DM1 patients are similar to those in patients with NPH. Furthermore, we found that DESH was observed in 20.6% of elderly patients with DM1 in our study. Previously, five cases with NPH in association with DM1 have been reported [12]. Three of those five patients received shunt surgery, and their condition markedly improved. These cases may support our results, as all of the cases were more than 50 years old, suggesting that DM1 patients may develop an NPH pathology later in life. Although cognitive deficits were found in a high percentage of DM1 patients at a late stage [6, 29, 30], the exact mechanism remains unclear. Therefore, further prospective studies with a larger number of DM1 patients are needed to determine whether or not an NPH-like appearance is associated with neuropsychological deficits.

The etiology and pathophysiology of the NPH-like appearance in DM1 are uncertain. One possible mechanism suspected by previous investigators involves widespread cell membrane defects caused by genetic abnormalities in DM1 [12, 31], leading to arachnoid granulations [32]. Another possible mechanism may be glymphatic system dysfunction in DM1. The glymphatic system hypothesized as a waste clearance pathway that removes metabolic wastes and neurotoxins from the brain along perivascular channels [33]. Recently, a MRI study disclosed that NPH patients have reduced glymphatic function along with enhanced accumulation and decreased clearance of an intrathecally administered contrast agent, gadobutrol [34]. The gadobutrol propagated at the brain surface along large leptomeningeal arteries. The cerebral metabolism was reduced in NPH [35]. More recent study showed that perivascular aquaporin‐4 (AQP4) expression is attenuated in NPH, potentially contributing to impaired glymphatic circulation, and waste clearance, and subsequent neurodegeneration [36]. The anatomical pathways for the glymphatic system are the perivascular spaces. While the perivascular spaces terminate within the brain parenchyma, paravascular cerebrospinal fluid (CSF) can continue traveling along the basement membranes surrounding arterial vascular smooth muscle [37]. In a previous neuropathological study, DM1 brain showed EPVS in the basal forebrain and deep white matter along with a loss of smooth muscles in small to medium-sized arteries [38]. A previous MRI study also showed that EPVS were significantly more frequent in DM1 patients (67%) than in controls (27%) [7]. Based on these previous findings, we suspect that the EPVS and NPH-like appearance in DM1 might reflect a pathology of glymphatic system dysfunction. On the other hand, we also found that z-Evans index was not associated with EPVS. Although the reason is unclear, brain compression due to ventriculomegaly might cause the obscuration of perivascular spaces. Furthermore, in previous studies, the EPVS increased in number with advancing age [39], patients with cerebral small vessel disease [40], patients with brain atrophy [41], or patients with hypertensive arteriopathy [39]. These results may suggest that EPVS might be caused by not only glymphatic system dysfunction but also various conditions.

For diagnosis of NPH, the clinical evaluation still often relies on the clinical presentation and morphologic findings on neuroimaging only, without assessment of the intracranial pressure, nor the actual CSF manometric profile. Therefore, the exact pathophysiological mechanism of NPH remains unclear. In previous review article, Bret et al. proposed a more relevant nosographic designation for the NPH syndrome by renaming it "chronic hydrocephalus" without reference to age and CSF pressure [42]. They also suggested that the "chronic hydrocephalus" is an active process that should be replaced in the sequence of events that result from failure of the circulatory resorptive mechanisms of the CSF, and it may be regarded as an intermediate state of balance between uncompensated hypertensive hydrocephalus and asymptomatic hydrocephalus. More recently, Taoka et al. proposed “CNS Interstitial Fluidopathy” as a new concept encompassing diseases whose pathologies are majorly associated with abnormal interstitial fluid dynamics [43]. In the “CNS interstitial fluidopathy”, various diseases or disorders, such as sleep disorders, Alzheimer’s disease, Parkinson’s disease, small vessel diseases of CNS, glaucoma, and NPH, were included. Moreover, according to its etiology, various conditions, mechanical force, abnormal blood–brain barrier, neuro-inflammation, abnormal distribution of hemorrhagic products, or abnormal vessel wall were categorized. Therefore, the DM1 with abnormal vessel wall in the brain parenchyma, a loss of smooth muscles in arteries [38], may also be included in the “CNS interstitial fluidopathy”.

Costanzo et al. showed a significant negative correlation between the degree of EPVS and disease duration and suggested that EPVS might progressively disappear during the disease course [7]. Our results regarding the patient age also showed that the presence of CS-EPVS was more common in Age-group A than in Age-group B and C, although there were no significant differences. Furthermore, regarding the frequency of temporo-polar WMLs, we found no significant differences among the age groups. These results suggest that CS-EPVS and/or temporo-polar WMLs may be findings at the early stage of DM1. Most of the elderly patients (Age-group C) with ventriculomegaly showed Sylvian fissure dilation, whereas only half of the younger patients showed this finding (Age-group A). In addition, we found that all patients with high-convexity tightness had ventriculomegaly as well as Sylvian fissure dilation. These results suggest that ventriculomegaly might precede the development of Sylvian fissure dilation and high-convexity tightness in DM1.

In DM1, the classic disease range of CTG repeat numbers is 50–4000, with repeat sizes of 50–80 associated with mild clinical phenotypes, while large repeat expansions of up to 4000 are often found in severe, mostly congenital forms of the disorder [44]. Brain abnormalities, including issues with cognitive function and muscle weakness, have been linked to the CTG repeat numbers, cognitive function, and muscle weakness [44–46]. However, the present study showed no correlation between the z-Evans Index and the CTG repeat numbers. Although the reason for this finding remains unclear, one possible reason is the small sample size of elderly patients with large repeat expansions because of their poor prognosis. Furthermore, our negative result may be supported by the findings of previous studies; for example, Minnerop et al. noted no correlations between neuropsychological test results and CTG repeat numbers [44]. With regard to neuroimaging assessments, temporo-polar WMLs have shown no correlation with CTG repeat numbers [7].

This study was limited by its retrospective design. Thus, neuropsychological tests were not performed in many cases, as neuropsychological deficits in cases of myotonic dystrophy can escape commonly applied neuropsychological test batteries for as-yet-unclear reasons. Moreover, no patients undergo lumbar puncture and were clinically diagnosed as having NPH. Second, our study included many qualitative analyses with subjective criteria. We arbitrarily defined ventriculomegaly as a z-Evans index of > 0.3 because an NPH-like appearance in DM1 patients is a relatively new finding.

## Conclusions

Our MRI study revealed an NPH-like appearance as a morphologic finding accompanied by ventriculomegaly in DM1 that tends to occur in elderly patients. Furthermore, because cognitive deficits are found in a high percentage of DM1 patients at the late stage, correlation analyses between neuropsychological deficits and the NPH-like appearances might be an interesting topic for a further study involving more patients.

## Data Availability

The datasets during and/or analysed during the current study available from the corresponding author on reasonable request.

## Abbreviations

DM1: Myotonic dystrophy type 1
NPH: Normal pressure hydrocephalus
DESH: Disproportionately enlarged subarachnoid-space hydrocephalus
EPVS: Enlarged perivascular spaces
CS: Centrum semiovale

## References

[1] Udd B, Krahe R (2012). The myotonic dystrophies: molecular, clinical, and therapeutic challenges. The Lancet Neurology.

[2] Nomenclature N, 1 Dtgfmdt: The International Myotonic Dystrophy Consortium (IDMC). Neurology 2000; 54:1218–1221. 10.1212/wnl.54.6.1218 10746587

[3] Consortium IMD (2000). New nomenclature and DNA testing guidelines for myotonic dystrophy type 1 (DM1). Neurology.

[4] Di Costanzo A, Di Salle F, Santoro L, Bonavita V, Tedeschi G (2001). T2 relaxometry of brain in myotonic dystrophy. Neuroradiology.

[5] Miaux Y, Chiras J, Eymard B, Lauriot-Prevost M, Radvanyi H, Martin-Duverneuil N, Delaporte C (1997). Cranial MRI findings in myotonic dystrophy. Neuroradiology.

[6] Huber SJ, Kissel JT, Shuttleworth EC, Chakeres DW, Clapp LE, Brogan MA (1989). Magnetic resonance imaging and clinical correlates of intellectual impairment in myotonic dystrophy. Arch Neurol.

[7] Di Costanzo A, Di Salle F, Santoro L, Tessitore A, Bonavita V, Tedeschi G (2002). Pattern and significance of white matter abnormalities in myotonic dystrophy type 1: an MRI study. J Neurol.

[8] Watson D, Hubbard B (1996). Adaptational style and dispositional structure: Coping in the context of the Five-Factor model. J Pers.

[9] Shprecher D, Schwalb J, Kurlan R (2008). Normal pressure hydrocephalus: diagnosis and treatment. Curr Neurol Neurosci Rep.

[10] Hashimoto M, Ishikawa M, Mori E, Kuwana N (2010). Improvement SoIoN: diagnosis of idiopathic normal pressure hydrocephalus is supported by MRI-based scheme: a prospective cohort study. Cerebrospinal Fluid Res.

[11] Di Costanzo A, Di Salle F, Santoro L, Bonavita V, Tedeschi G (2002). Brain MRI features of congenital-and adult-form myotonic dystrophy type 1: case-control study. Neuromuscul Disord.

[12] Wang J, Liu M, Shang W, Chen Z, Peng G (2020). Myotonic dystrophy type 1 accompanied with normal pressure hydrocephalus: a case report and literature review. BMC Neurol.

[13] Brook JD, McCurrach ME, Harley HG, Buckler AJ, Church D, Aburatani H, Hunter K, Stanton VP, Thirion J-P, Hudson T (1992). Molecular basis of myotonic dystrophy: expansion of a trinucleotide (CTG) repeat at the 3′ end of a transcript encoding a protein kinase family member. Cell.

[14] Yamada S, Ishikawa M, Yamamoto K (2015). Optimal diagnostic indices for idiopathic normal pressure hydrocephalus based on the 3D quantitative volumetric analysis for the cerebral ventricle and subarachnoid space. Am J Neuroradiol.

[15] Virhammar J, Laurell K, Cesarini KG, Larsson E-M (2014). Preoperative prognostic value of MRI findings in 108 patients with idiopathic normal pressure hydrocephalus. Am J Neuroradiol.

[16] Narita W, Nishio Y, Baba T, Iizuka O, Ishihara T, Matsuda M, Iwasaki M, Tominaga T, Mori E (2016). High-convexity tightness predicts the shunt response in idiopathic normal pressure hydrocephalus. Am J Neuroradiol.

[17] Miyata M, Kakeda S, Iwata S, Nakayamada S, Ide S, Watanabe K, Moriya J, Tanaka Y, Korogi Y (2017). Enlarged perivascular spaces are associated with the disease activity in systemic lupus erythematosus. Sci Rep.

[18] Farrell C, Chappell F, Armitage P, Keston P, MacLullich A, Shenkin S, Wardlaw J (2009). Development and initial testing of normal reference MR images for the brain at ages 65–70 and 75–80 years. Eur Radiol.

[19] Charidimou A, Jaunmuktane Z, Baron J-C, Burnell M, Varlet P, Peeters A, Xuereb J, Jäger R, Brandner S, Werring DJ (2014). White matter perivascular spaces: an MRI marker in pathology-proven cerebral amyloid angiopathy?. Neurology.

[20] Team RC: R: A language and environment for statistical computing. R Foundation for Statistical Computing, Vienna, Austria. 2013. In*.*: ISBN 3-900051-07-0; 2014.

[21] Evans WA (1942). An encephalographic ratio for estimating ventricular enlargement and cerebral atrophy. Arch Neuro Psychiatry.

[22] Virhammar J, Laurell K, Cesarini KG, Larsson E-M (2014). The callosal angle measured on MRI as a predictor of outcome in idiopathic normal-pressure hydrocephalus. J Neurosurg.

[23] Ishii K, Kanda T, Harada A, Miyamoto N, Kawaguchi T, Shimada K, Ohkawa S, Uemura T, Yoshikawa T, Mori E (2008). Clinical impact of the callosal angle in the diagnosis of idiopathic normal pressure hydrocephalus. Eur Radiol.

[24] Yamada S, Ishikawa M, Yamaguchi M, Yamamoto K (2019). Longitudinal morphological changes during recovery from brain deformation due to idiopathic normal pressure hydrocephalus after ventriculoperitoneal shunt surgery. Sci Rep.

[25] Virhammar J, Laurell K, Cesarini KG, Larsson E-M (2018). Increase in callosal angle and decrease in ventricular volume after shunt surgery in patients with idiopathic normal pressure hydrocephalus. J Neurosurg.

[26] Kockum K, Lilja-Lund O, Larsson EM, Rosell M, Söderström L, Virhammar J, Laurell K (2018). The idiopathic normal-pressure hydrocephalus Radscale: a radiological scale for structured evaluation. Eur J Neurol.

[27] Shinoda N, Hirai O, Hori S, Mikami K, Bando T, Shimo D, Kuroyama T, Kuramoto Y, Matsumoto M, Ueno Y (2017). Utility of MRI-based disproportionately enlarged subarachnoid space hydrocephalus scoring for predicting prognosis after surgery for idiopathic normal pressure hydrocephalus: clinical research. J Neurosurg.

[28] Cagnin A, Simioni M, Tagliapietra M, Citton V, Pompanin S, Della Puppa A, Ermani M, Manara R (2015). A simplified callosal angle measure best differentiates idiopathic-normal pressure hydrocephalus from neurodegenerative dementia. J Alzheimers Dis.

[29] Meola G, Sansone V, Perani D, Colleluori A, Cappa S, Cotelli M, Fazio F, Thornton C, Moxley R (1999). Reduced cerebral blood flow and impaired visual–spatial function in proximal myotonic myopathy. Neurology.

[30] Modoni A, Silvestri G, Pomponi MG, Mangiola F, Tonali PA, Marra C (2004). Characterization of the pattern of cognitive impairment in myotonic dystrophy type 1. Arch Neurol.

[31] Christensen PB (1988). Normal pressure hydrocephalus in myotonic dystrophy. Eur Neurol.

[32] Duinkerke A, Williams MA, Rigamonti D, Hillis AE (2004). Cognitive recovery in idiopathic normal pressure hydrocephalus after shunt. Cogn Behav Neurol.

[33] Jessen NA, Munk ASF, Lundgaard I, Nedergaard M (2015). The glymphatic system: a beginner’s guide. Neurochem Res.

[34] Ringstad G, Vatnehol SAS, Eide PK (2017). Glymphatic MRI in idiopathic normal pressure hydrocephalus. Brain.

[35] Calcagni ML, Taralli S, Mangiola A, Indovina L, Lavalle M, De Bonis P, Anile C, Giordano A (2013). Regional cerebral metabolic rate of glucose evaluation and clinical assessment in patients with idiopathic normal-pressure hydrocephalus before and after ventricular shunt placement: a prospective analysis. Clin Nucl Med.

[36] Hasan-Olive MM, Enger R, Hansson HA, Nagelhus EA, Eide PK (2019). Loss of perivascular aquaporin-4 in idiopathic normal pressure hydrocephalus. Glia.

[37] Rennels ML, Gregory TF, Blaumanis OR, Fujimoto K, Grady PA (1985). Evidence for a ‘paravascular’fluid circulation in the mammalian central nervous system, provided by the rapid distribution of tracer protein throughout the brain from the subarachnoid space. Brain Res.

[38] Itoh K, Mitani M, Kawamoto K, Futamura N, Funakawa I, Jinnai K, Fushiki S: Neuropathology does not correlate with regional differences in the extent of expansion of CTG repeats in the brain with myotonic dystrophy type 1. Acta histochemica et cytochemica 2010:1012160093–1012160093. 10.1267/ahc.10019 PMC3015052 21245981

[39] Zhu Y-C, Tzourio C, Soumaré A, Mazoyer B, Dufouil C, Chabriat H (2010). Severity of dilated Virchow-Robin spaces is associated with age, blood pressure, and MRI markers of small vessel disease: a population-based study. Stroke.

[40] Doubal FN, MacLullich AM, Ferguson KJ, Dennis MS, Wardlaw JM (2010). Enlarged perivascular spaces on MRI are a feature of cerebral small vessel disease. Stroke.

[41] Zhang X, Ding L, Yang L, Qin W, Yuan J, Li S, Hu W (2016). Brain atrophy correlates with severe enlarged perivascular spaces in basal ganglia among lacunar stroke patients. PLoS ONE.

[42] Bret P, Guyotat J, Chazal J (2002). Is normal pressure hydrocephalus a valid concept in 2002? A reappraisal in five questions and proposal for a new designation of the syndrome as “chronic hydrocephalus”. J Neurol Neurosurg Psychiatry.

[43] Taoka T, Naganawa S (2020). Imaging for central nervous system (CNS) interstitial fluidopathy: disorders with impaired interstitial fluid dynamics. Jpn J Radiol.

[44] Minnerop M, Weber B, Schoene-Bake J-C, Roeske S, Mirbach S, Anspach C, Schneider-Gold C, Betz RC, Helmstaedter C, Tittgemeyer M (2011). The brain in myotonic dystrophy 1 and 2: evidence for a predominant white matter disease. Brain.

[45] Antonini G, Mainero C, Romano A, Giubilei F, Ceschin V, Gragnani F, Morino S, Fiorelli M, Soscia F, Di Pasquale A (2004). Cerebral atrophy in myotonic dystrophy: a voxel based morphometric study. J Neurol Neurosurg Psychiatry.

[46] Weber YG, Roebling R, Kassubek J, Hoffmann S, Rosenbohm A, Wolf M, Steinbach P, Jurkat-Rott K, Walter H, Reske SN (2010). Comparative analysis of brain structure, metabolism, and cognition in myotonic dystrophy 1 and 2. Neurology.

